# Supplementary material for: PromarkerD Predicts Renal Function Decline in Type 2 Diabetes in the Canagliflozin Cardiovascular Assessment Study (CANVAS)
Source: J Clin Med. 2020 Oct 6;9(10):3212. doi: 10.3390/jcm9103212 (PMC7600174; doi:10.3390/jcm9103212)
Supplement: Supplementary file 1 [file jcm-09-03212-s001.pdf]

Table S1. PromarkerD Scores at baseline (continuous and risk categories) by treatment arm.

PromarkerD Scores	PBO	CANA	*p*-value
Prognostic (Incident CKD) *			
Number of subjects	982	1994	
Mean ± SD	8.7 ± 12.3	9.0 ± 12.6	0.50
Range	0.0–73.1	0.0–66.8	
Geometric Mean (SD range)	1.9 (0.2–20.7)	2.0 (0.2–20.7)	0.51
Median (IQR)	2.7 (0.5–12.6)	2.9 (0.5–12.9)	0.56
Low-Risk (%)	691 (70.4)	1408 (70.6)	0.24
Moderate-Risk (%)	146 (14.9)	259 (13.0)	
High-Risk (%)	145 (14.8)	327 (16.4)	
Prognostic (Decline ≥30%) *			
Number of subjects	1179	2346	
Mean ± SD	2.8 ± 2.4	2.9 ± 2.4	0.58
Range	0.0–18.4	0.0–19.5	
Geometric Mean (SD range)	1.8 (0.6–5.6)	1.9 (0.6–5.6)	0.45
Median (IQR)	2.2 (1.2–3.8)	2.3 (1.2–3.9)	0.59
Low-Risk (%)	1157 (98.1)	2307 (98.3)	0.68
Moderate-Risk (%)	22 (1.9)	39 (1.7)	
High-Risk (%)	N/A †	N/A †	
Diagnostic (Baseline CKD)			
Number of subjects	1190	2361	
Mean ± SD	39.0 ± 14.1	39.8 ± 14.0	0.15
Range	3.6–74.2	3.3–74.4	
Median (IQR)	40.0 (30.0–48.9)	40.4 (31.0–49.8)	0.17
Low-Risk (%)	299 (25.1)	543 (23.0)	0.12
Moderate-Risk (%)	824 (69.2)	1649 (69.8)	
High-Risk (%)	67 (5.6)	169 (7.2)	

Only participants with PromarkerD scores and outcome data are shown. Independent samples *t*-test for means and geometric means; Independent samples Kruskal-Wallis Test for medians. * The two prognostic scores were ln-transformed due to a non-normal distribution. † No participants were observed in the Prognostic (Decline ≥30%) high-risk category.

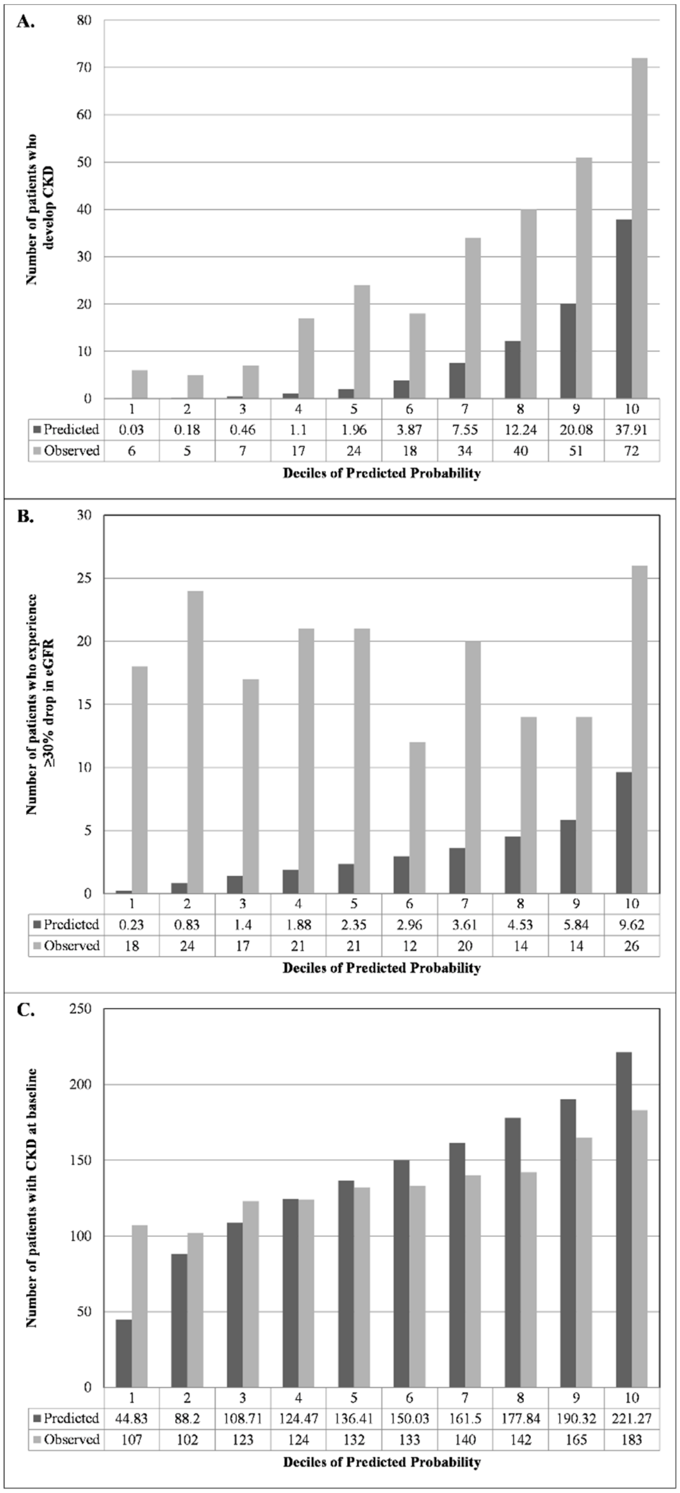

Figure S1. Calibration plots showing the observed and predicted number of subjects experiencing each renal outcome by deciles of risk in participants from the placebo arm of the CANVAS trial. A. Incident CKD, B. eGFR decline ≥30%, C. CKD at baseline (placebo plus canagliflozin). Observed numbers are shown by the light grey bars and predicted numbers by the dark grey bars.

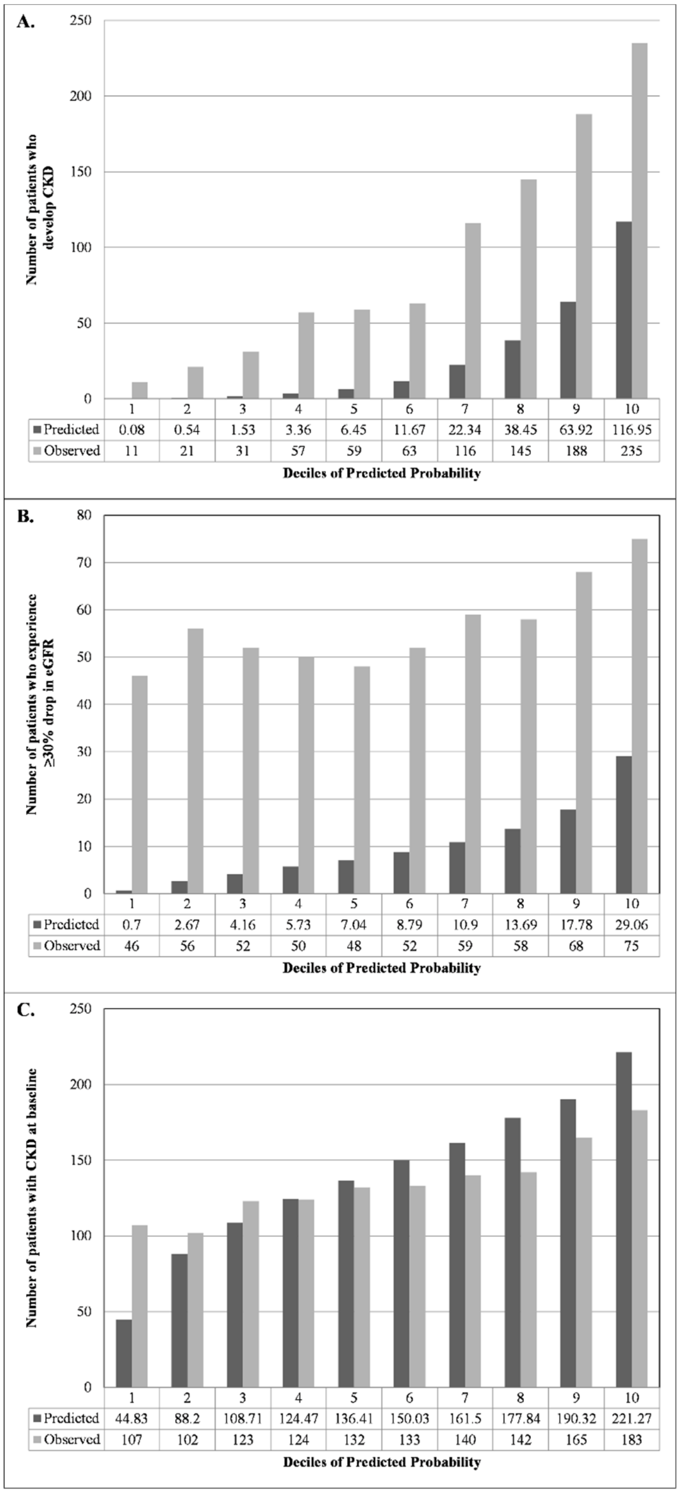

Figure S2. Calibration plots showing the observed and predicted number of subjects experiencing each renal outcome by deciles of risk in all participants (placebo and canagliflozin arms combined) of the CANVAS trial. A. Incident CKD, B. eGFR decline ≥30%, C. CKD at baseline. Observed numbers are shown by the light grey bars and predicted numbers by the dark grey bars.
